# The inactivated and ISA 61 VG adjuvanted vaccine enhances protection against cross-serotype *Listeria monocytogenes*

**DOI:** 10.1186/s13567-025-01483-2

**Published:** 2025-03-20

**Authors:** Fanzeng Meng, Ye Wang, Chao Chen, Tianxiang Pan, Jing Li, Yao Xu, Zegang Wang, Hao Yao, Xin’an Jiao, Yuelan Yin

**Affiliations:** 1 Jiangsu Key Laboratory of Zoonosis, Yangzhou University, Yangzhou, 225009 Jiangsu China; 2 Key Laboratory of Prevention and Control of Biological Hazard Factors (Animal Origin) for Agrifood Safety and Quality, Ministry of Agriculture of China, Yangzhou University, Yangzhou, 225009 Jiangsu China; 3 Joint International Research Laboratory of Agriculture and Agri-Product Safety of the Ministry of Education, Yangzhou, 225009 Jiangsu China; 4 Jiangsu Co-Innovation Center for Prevention and Control of Important Animal Infectious Diseases and Zoonoses, Yangzhou, 225009 Jiangsu China

**Keywords:** *Listeria monocytogenes* (Lm), adjuvanted inactivated vaccine (AIV), ISA 61 VG adjuvant, cross-immunoprotection, sheep

## Abstract

Listeriosis is a zoonotic disease caused by *Listeria monocytogenes* (Lm), posing a significant threat to the breeding industry and public health. Ruminant livestock are particularly susceptible to Lm,  thus effective strategies are needed for controlling ovine listeriosis. In this study, we developed two inactivated vaccines and evaluated their efficacy against Lm infection in murine and ovine models. We inactivated the Lm serotype 4h XYSN strain and adjuvanted it with water-in-oil ISA 61 VG (61 VG-AIV) or aluminum (Al-AIV). Pathological observations confirmed the safety of both vaccines in mice and sheep. The immunological assays demonstrated that, compared with the Al-AIV, the 61 VG-AIV induced higher levels of Lm-specific antibodies and proinflammatory cytokines, suggesting that the ISA 61 VG adjuvant has superior immunostimulatory effects compared with the alum adjuvant. 61 VG-AIV elicited greater immunoprotection than Al-AIV (83.4% vs. 50%) against serotype 4h Lm strain challenge in mice. Additionally, 61 VG-AIV afforded cross-protection against challenges with serotypes 1/2a, 1/2b, and 4b Lm strains. Importantly, high immunoprotection in sheep was conferred by the 61 VG-AIV group (83.4%). Taken together, our findings demonstrate that the ISA 61 VG adjuvant contributes to enhancing the humoral and cellular immune responses of inactivated Lm, and 61 VG-AIV is a promising vaccine candidate for the prevention and control of animal listeriosis. This research lays a solid foundation for its application in veterinary medicine.

**Supplementary Information:**

The online version contains supplementary material available at 10.1186/s13567-025-01483-2.

## Introduction

*Listeria monocytogenes* (Lm) is a Gram-positive zoonotic pathogen that can infect more than 60 species of animals, including humans, ruminants, rodents, birds, fish and molluscs. Ruminant animals, particularly sheep, are most susceptible to Lm. *Listeria* cells enter the gut of sheep through contaminated feed or water and then break the intestinal barrier, the blood‒brain barrier or the blood‒foetal barrier, causing septicaemia, gastroenteritis, meningitis and ewe miscarriage. The sporadic ovine outbreak of listeriosis poses a serious threat not only to the breeding industry but also to human health and public safety. Human listeriosis is manifested by symptoms such as sepsis, meningitis, abortion, and fatality, with a mortality rate of 25–30%. Listeriosis outbreaks, significant public health threats, are frequently reported [1]. The 2018 listeriosis outbreak in South Africa recorded a total of 934 confirmed cases, ultimately resulting in 193 deaths [2]. Antibiotics are commonly employed against clinical listeriosis; however, concerns about antibiotic resistance in Lm strains are increasing [3]. Vaccination is crucial for the prevention and treatment of listeriosis. To date, there are no commercially available vaccines for human or animal listeriosis. Therefore, there is an urgent need to develop effective vaccines against *Listeria* infections to reduce the threat to public health and the livestock industry.

Lm can transit from a saprophytic state to an intracellular state following its ingestion in the gut of the host. A variety of virulence factors are involved in the adaptation of *Listeria* to intracellular life. The virulence factors listeriolysin O, PlcA and PlcB help Lm escape from the phagolysosome to the host cell cytoplasm; therefore, *Listeria* antigens are processed and presented to CD8^+^ T cells and CD4^+^ T cells via MHC-I or MHC-II complexes [4]. Owing to its inherent parasitic nature, the elimination of Lm within the host mainly depends on cellular immune responses, and humoral immune responses also play important roles in defending against intracellular pathogens [5]. Live attenuated Lm vaccines, which are achieved through the targeted deletion of bacterial virulence genes, are safe for the host while retaining the ability to elicit substantial cellular immune responses [6, 7]. Inactivated vaccines or subunit vaccines combined with specific adjuvants dramatically improve safety and efficiently confer protective immunity. Adjuvants play a critical role in enhancing the immunity of various types of vaccines. Inactivated pathogens exhibit a limited ability to induce cellular immune responses, so the combination of adjuvants is essential for enhancing immunogenicity and increasing the duration of antigen stimulation [8]. Common adjuvants include aluminium adjuvants and oil-based adjuvants. Notably, aluminium adjuvants tend to induce Th2-type immune responses and are less effective at eliciting cellular immunity [9]. Freund’s adjuvant, a mineral oil-based oil-in-water adjuvant, is referred to as Freund’s complete adjuvant (CFA) or Freund’s incomplete adjuvant (IFA). CFA, which contains *Mycobacterium tuberculosis* cell wall components, can induce a Th1 immune response but may cause significant inflammation and discomfort at the site of inoculation [10, 11]. Recently, refined mineral oil emulsion adjuvants, such as water in oil (W/O), water-in-oil in water (W/O/W), and oil-in-water (O/W), have become available. Montanide^TM^ ISA 61 VG, a water-in-oil adjuvant, encapsulates bacterial antigens, thereby forming antigen reservoirs at the injection site. This design allows prolonged immune system stimulation, resulting in elevated antibody levels and the induction of a Th1-biased immune response [12, 13].

Small ruminants, particularly sheep and goats, are naturally susceptible hosts to *Listeria* [14, 15]. ISA 61 VG has been used as a vaccine adjuvant in the development of inactivated ruminant vaccines [16, 17]. Various small ruminant vaccine formulations using attenuated Lm have been immunologically evaluated in the past few decades [18, 19]. Some live attenuated *Listeria* vaccines have shown reduced bacterial loads and varying degrees of protection against *Listeria* infection in small ruminants [19, 20]. These studies have identified promising candidate vaccines that have inspired further research into *Listeria* vaccines for small ruminants. In a previous study, a candidate live vaccine increased the survival rate of vaccinated sheep by 78% compared with that of the unvaccinated group following Lm challenge [19]. However, the use of live attenuated vaccines in food-producing animals is limited due to safety and regulatory issues.

Currently, no commercially available vaccines exist for human or animal listeriosis. The hypervirulent serotype 4h Lm has caused multiple ovine listeriosis outbreaks. Lm XYSN, which has unique glycosylated wall teichoic acids, was isolated from the brains of sick goats [21]. In this study, we developed an inactivated serotype 4h Lm vaccine adjuvanted with ISA 61 VG (61 VG-AIV). We demonstrated the safety, immunogenicity, and cross-protection of 61 VG-AIV against four Lm serotypes in a mouse model. In addition we employed the sheep model for evaluation the safety, immunogenicity, and protective efficacy of this vaccine were further identified evaluated in a sheep model. This research aims to understand the potential of 61 VG-AIV for preventing and controlling Lm infection in ruminants.

## Materials and methods

### Bacterial strains and animals

The virulent Lm strains EGD-e (serotype 1/2a), Yc32 (serotype 1/2b), NTSN (serotype 4b), and XYSN (serotype 4h) are stored at the Jiangsu Key Laboratory of Zoonosis. Six-week-old female C57BL/6N mice were purchased from Charles River Experimental Animals Co., Ltd., and 3-month-old sheep were purchased from Suzhou. The study strictly adhered to the recommendations outlined in the Laboratory Animals: A Guide to the Use of Animals in Education by the China Association for Laboratory Animal Sciences (CALAS). Approval for the animal study was granted by the Animal Ethics Committee of Yangzhou University (No. 202106009 and No. 202211018).

### Preparation and immunization of 61 VG-AIV

**Figure 1:**
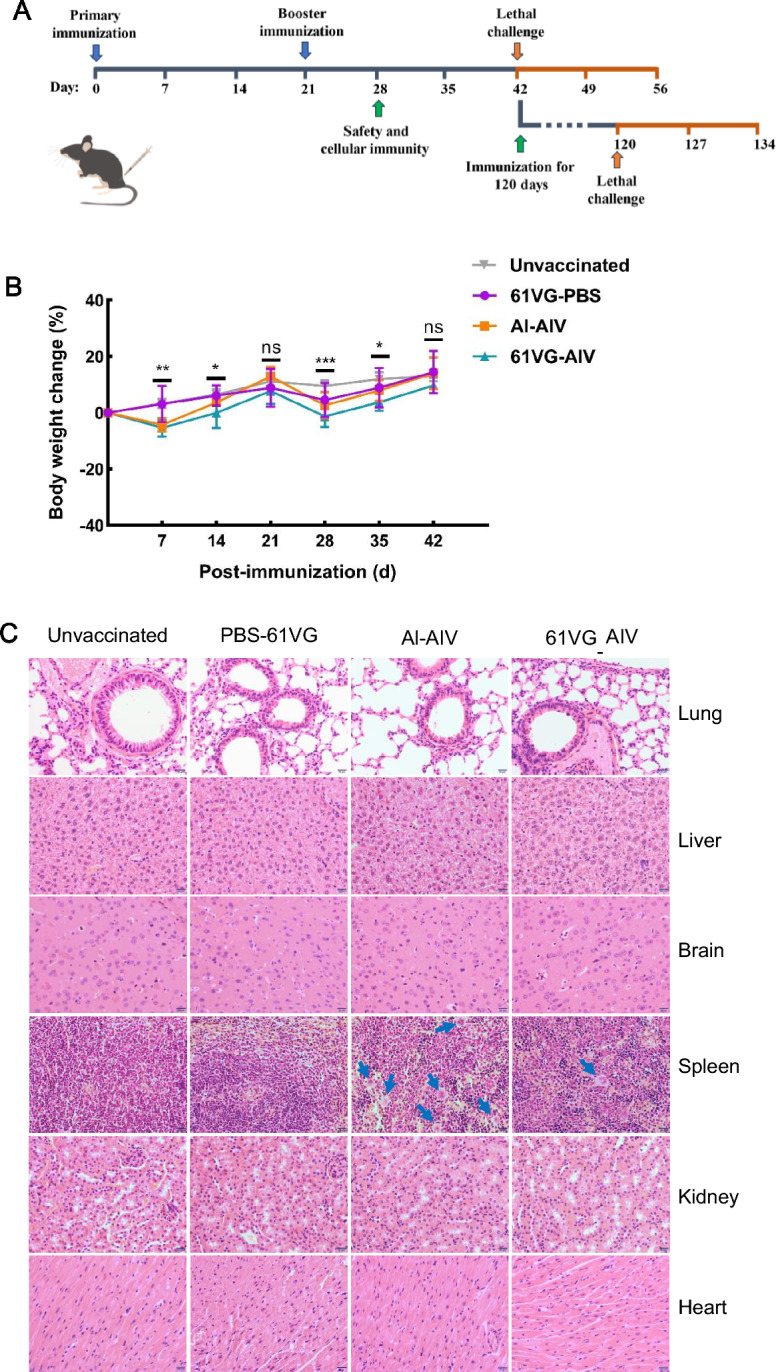
**Safety of 61VG-AIV in mice**. **A** C57BL/6N mice were initially immunized on day 0 and received a booster immunization on day 21. These mice were then challenged with lethal doses of the wild-type strain Lm either intraperitoneally or orally 21 days after booster immunization. Blood samples were collected from the mice on days 7, 14, 21, 28, 35, 42, and 49 post-immunization, and the serum was harvested. The survival of C57BL/6N mice challenged with Lm was monitored for 14 days (*n* = 6). **B** C57BL/6N mice were divided into three groups (*n* = 6). The weights of the mice were measured with an electronic balance after vaccination at 7-day intervals. The figure shows the percentage of weight gain (or loss) for each mouse. The error bars represent the SD; *n* = 3 independent experiments. Statistical analyses were carried out using unpaired t tests: **P < 0.01, *P < 0.05, ns ≥ 0.05, compared with the corresponding control group. **C** Histopathological sections of vaccinated mouse organs were observed and analysed via HE staining of tissue sections (lung, liver, brain, spleen, kidney, and heart). Series 1) shows the organs of the mice in the blank group; 2) the organs of the mice in the PBS group; 3) the organs of the mice in the Al-AIV group; and 4) the organs of the mice in the 61 VG-AIV group. In the spleen, more macrophages infiltrated the mice in the Al-AIV group (blue arrow), and a small amount of macrophages infiltrated the mice in the ISA 61 VG adjuvant group (blue arrow). Images were taken at 400 × magnification. No obvious pathological changes were found in other organs.

**Figure 2:**
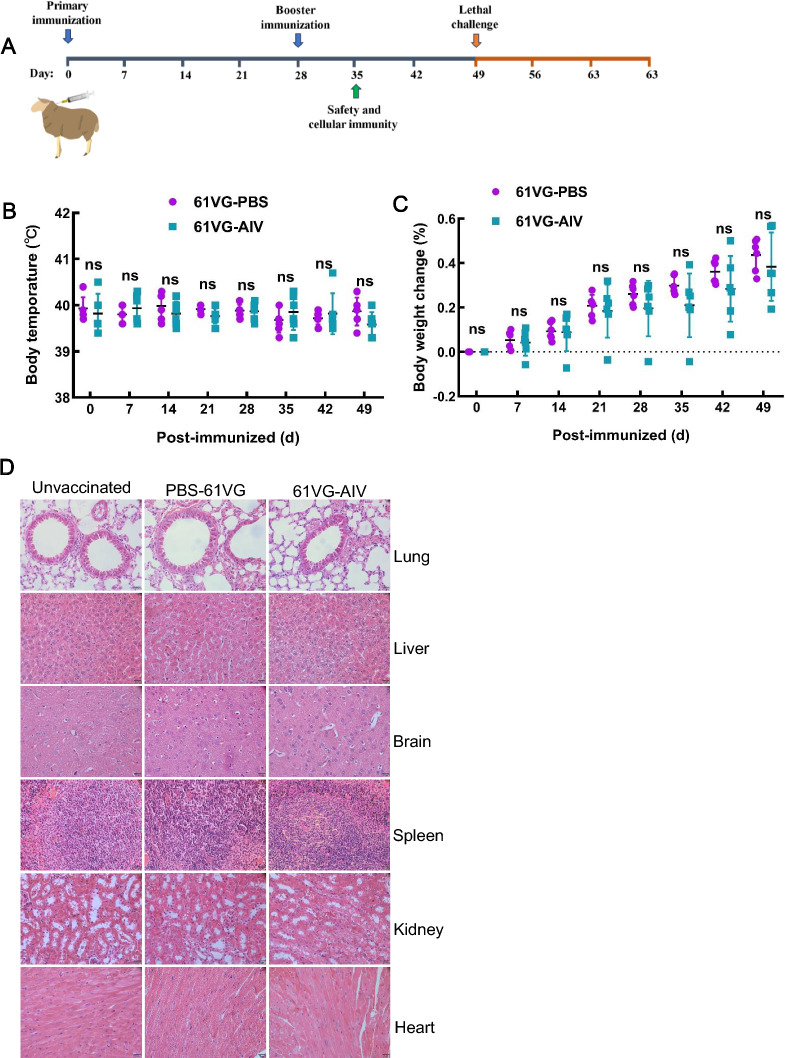
**Safety of 61 VG-AIV in sheep**. **A** The sheep were vaccinated on day 0 and received a second vaccination on day 28. The sheep were subsequently challenged intramuscularly at 28 days after booster immunization. Blood samples from the sheep were collected at 7, 21, 35, and 49 days after immunization, and the serum was harvested. The survival of the challenged sheep was recorded daily for 21 days (*n* = 6). **B** and** C** Sheep were divided into two groups (*n* = 6). The rectal temperature and weight of the sheep were measured after vaccination at 7-day intervals. The error bars represent the SD. Statistical analyses were performed using unpaired t tests: **P < 0.01, *P < 0.05, ns ≥ 0.05, compared with the 61 VG-AIV group. **D** Series 1) shows the organs of unvaccinated sheep; 2) the organs of the 61 VG-PBS sheep; and 3) the organs of the 61 VG-AIV sheep. Micrographs of hematoxylin and eosin (H&E)-stained sections of the heart, liver, spleen, lung, kidney, and brain of sheep immunized with 61 VG-AIV and the control group 61 VG-PBS; the unvaccinated group was used as a control. Images were taken at 400 × magnification. Pathology of various organs in the 61 VG-AIV- and 61 VG-PBS-inoculated groups revealed no inflammatory cell infiltration, necrosis, or fibrosis.

The Lm XYSN strain was cultured in brain heart infusion broth for 12–16 h and then expanded at a 1:30 ratio for 3 h. The bacteria were subsequently washed twice with PBS, followed by the addition of formaldehyde to a final concentration of 0.4% to the bacterial suspension at a concentration of 1 × 10^9^ CFU/mL. The bacterial suspension was subsequently incubated at 37 °C for 24 h and shaken at 1–2-h intervals to prevent bacterial sedimentation. Next, the bacterial suspension was spread on BHI plates and inoculated into BHI liquid media to check for complete inactivation. The ISA 61 VG-adjuvant inactivated vaccine (abbreviated as 61 VG-AIV) was prepared by emulsifying a 35% bacterial suspension in 65% Montanide™ ISA 61 VG-adjuvant (Seppic Chemical Specialities Co., Ltd., Shanghai, China). An aluminum adjuvant (Thermo Fisher Scientific, USA) was mixed at a 1:1 ratio with the bacterial suspension to obtain the inactivated aluminum adjuvant inactivated vaccine (Al-AIV). The mice received prime-boost immunization with ISA 61 VG (61 VG-PBS), Al-AIV, or 61 VG-AIV on day 1 and on day 21 via the intramuscular (i.m.) route (Table 1). The mice in each group were challenged on day 21 after booster immunization (Figures 1A, 2A). The inoculation dose of the inactivated vaccine was 1 × 10^10^ CFU/mouse in a 200 μL volume. The sheep were administered 1 × 10^10^ CFU in 1.5 mL via subcutaneous immunization.

**Table 1:** **Animal experiment grouping information**

Animal species	Groups name	Components	Antigen dose/CFU	Volumes
Mice	61 VG-PBS	ISA 61 VG and PBS	–	200 μL
Mice	IV	Inactivated XYSN	1 × 10^10^	200 μL
Mice	Al-AIV	Alum adjuvant and inactivated XYSN	1 × 10^10^	200 μL
Mice	61 VG-AIV	ISA 61 VG and inactivated XYSN	1 × 10^10^	200 μL
Sheep	61 VG-PBS	ISA 61 VG and PBS	–	1.5 mL
Sheep	61 VG-AIV	and inactivated XYSN	1 × 10^10^	1.5 mL

### Safety of 61 VG-AIV

Safety was evaluated in both sheep and mouse models. First, we measured body weight and pathological changes in a mouse model. The weight changes of the mice were monitored on days 7, 14, 21, 28, 35, and 42, with a booster immunization administered on day 21 after the initial immunization. On day 7 after booster immunization, the heart, liver, spleen, lung, kidney, and brain were collected from the mice and fixed in 10% formaldehyde. Next, we observed the injection site and signs of the vaccinated animals and measured their body weight and rectal temperature in sheep, using electronic scales and thermometers. The safety assessment of 61 VG-PBS and 61 VG-AIV in sheep included weekly monitoring of body weight changes and rectal temperature following intramuscular inoculation. A booster immunization was conducted on day 28 after the initial immunization, and 7 days after booster immunization, one sheep randomly selected from each group was euthanized, and its organs (heart, liver, spleen, lung, kidney, and brain) were collected and fixed in 10% formaldehyde solution. The fixed organs from the mice and sheep were sectioned and subjected to haematoxylin‒eosin (H&E) staining at Servicebio Technology Co., Ltd. (Wuhan). Histopathological changes were observed by optical microscopy and photographed for analysis. The pathological scoring criteria are shown in Table 2.

**Table 2:** **Histopathology scores of the tissues from the mice and sheep**

Score	Neutrophils	Necrosis
0	No lesions	No lesions
1	Mild local neutrophil infiltration	Mild local necrosis and cell swelling
2	Mild to moderate local neutrophil infiltration	Mild to moderate local necrosis and cell swelling
3	Moderate local neutrophil infiltration	Moderate local necrosis and cell swelling
4	Severe local neutrophil infiltration	Severe local necrosis and cell swelling

### IgG and IgG subtype antibodies assays in mice

Humoral immune responses play a crucial role in eliminating Lm [22, 23]. Here, Lm-specific IgG antibodies were assessed by indirect ELISA. Three groups of mice (*n* = 6/group) received priming and booster immunizations with 61 VG-AIV, Al-AIV, or 61 VG-PBS to evaluate the humoral immune response. Blood samples were collected at days 0, 7, 14, 21, 28, 35, and 42 post-primary immunization for serum isolation. An indirect enzyme-linked immunosorbent assay (ELISA) was used to measure IgG antibody titres against Lm XYSN in the serum.

The ELISA procedure involved incubating a 5% glutaraldehyde solution at 37 ℃ for 2 h, followed by the addition of 50 μL of the Lm XYSN suspension (1.0 × 10^8^ CFU/mL) to each well to coat the Lm XYSN whole cells. Glutaraldehyde can effectively reduce the nonspecific adsorption of antigens to ELISA plates through covalent cross-linking, thereby improving the specificity of detection. Mouse sera were used as the primary antibodies for ELISA, subjected to gradient dilutions, and incubated in the wells of the assay plate. HRP-conjugated goat anti-mouse IgG, IgG1, and IgG2a antibodies (Sigma, USA) diluted at 1:5000 were added (100 μL) for incubation. The reaction was stopped by adding 2 N H_2_SO_4_ after adding TMB for 10 min. Absorbance at 450 nm (OD_450_) was measured with a multifunctional microplate reader, with a P (sample)/N (negative control) ratio ≥ 2.1 considered positive. The IgG1/IgG2a antibody titre ratio was determined to indicate Th1/Th2 immune reaction bias.

### Cytokine transcript levels in mouse splenocytes

On day 14 post-booster immunization, the spleens of four mice (*n* = 4) were collected, and total RNA extraction was performed using an animal total RNA extraction kit according to the manufacturer’s instructions (TianGen, Beijing). Subsequently, reverse transcription and cDNA synthesis were performed according to the manufacturer’s protocol (TAKARA, Dalian). The quantitative real-time polymerase chain reaction (qRT‒PCR) was performed via FSU SYBR Green Master (Rox) (Enzyme, Nanjing) on a 7500 Real-Time PCR System (Thermo Fisher, USA), with the primers listed in Table 3. The housekeeping gene GAPDH was used as an internal control for normalization of the experimental results. The relative quantity (RQ) of cytokine gene expression was determined via the 2^−ΔΔCT^ method.

**Table 3:** **Primer sequences for cytokines used in qRT-PCR**

Primers	Sequence (5'-3')
IL-4-F	TCACAGCAACGAAGAACACC
IL-4-R	CGAAAAGCCCGAAAGAGTC
IL-6-F	TACCACTCCCAACAGACC
IL-6-R	CATTTCCACGATTTCCCAGA
IL-17-F	AAACACTGAGGCCAAGGAC
IL-17-R	CGTGGAACGGTTGAGGTAG
TNF-α-F	TCTCATTCCTGCTTGTGG
TNF-α-R	ACTTGGTGGTTTGCTACGA
IFN-γ-F	AGGAACTGGCAAAAGGATGGT
IFN-γ-R	ACGCTTATGTTGTTGCTGATGG
GAPDH-F	CAAATTCAACGGCACAGTCA
GAPDH-R	TTAGTGGGGTCTCGCTCC

### Protective efficacy of 61VG-AIV in mice

This study investigated cross-protection against challenge with four serotypes of Lm following prime-boost vaccination of C57BL/6N mice with 61 VG-AIV. Each serotype was assessed in three groups of mice (*n* = 6/group) to evaluate cross-protection. On day 21 post-booster immunization, the mice were challenged with a lethal dose of different Lm serotypes (4h XYSN, 1/2a EGD-e, 1/2b Yc32, or 4b NTSN) (Table 4). Mouse survival was monitored for 14 days after the challenge, and survival curves were subsequently plotted.

**Table 4:** **Mouse challenge immunity protection experiment**

Lm serotype	Lineage	Challenge/CFU	Challenge procedure
1/2a	II	1.25 × 10^8^	Intraperitoneal
1/2b	I	1.25 × 10^7^	Intraperitoneal
4b	I	3.5 × 10^6^	Intraperitoneal
4 h	II	4.5 × 10^7^	Oral

Six-week-old female C57BL/6 mice (*n* = 5 per group) were randomly divided into five groups: PBS, 61 VG-PBS, AIV, Al-AIV, and 61 VG-AIV. Primer booster immunization was administered at two-week intervals. Two weeks after booster vaccination, the mice were orally challenged with a lethal dose of 1 × 10^7^ CFU of Lm XYSN. Bacterial loads in the spleen, liver, lungs, and brain were assessed on day 4 post-challenge. The tissue samples were homogenized, serially diluted, and plated on BHI agar plates to quantify the bacterial loads in each group. Another portion of the tissues was subjected to histopathological analysis, with sections stained with HE and scored according to a predefined scoring system (Table 2). Additionally, body weight changes were recorded at 0, 1, 2, 3, and 4 days post-infection.

### Duration of antibody response in 61 VG-AIV-immunized mice

**Figure 3:**
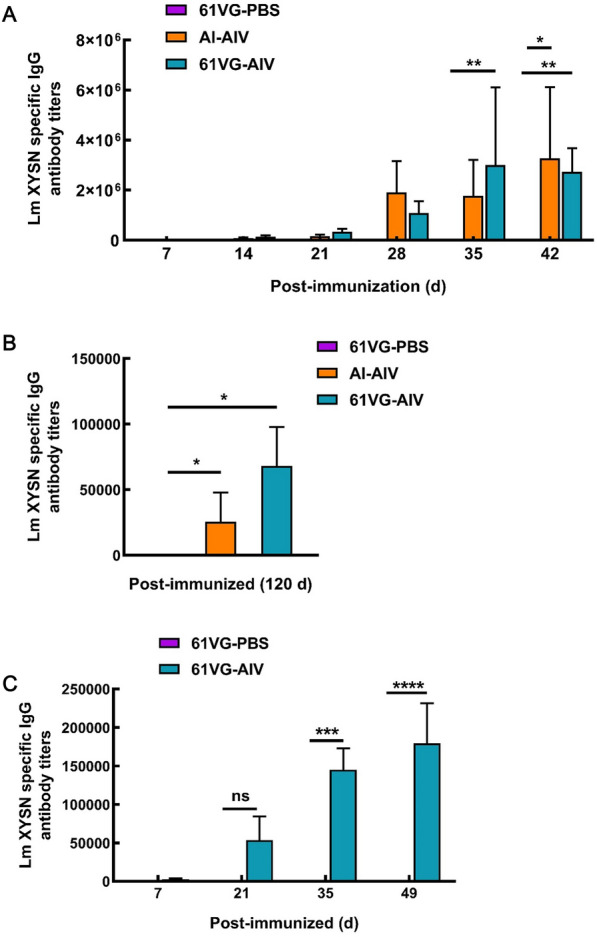
**Humoral immunity of immunized animal models**. **A** The level of IgG antibody in the serum of immunized mice. **B** Serum antibody titre on day 120 after immunization in the mice. **C** Changes in IgG antibody levels in sheep serum. The error bars represent the SD; *n* = 3 independent experiments. Statistical analyses were carried out via two-way ANOVA with Tukey’s multiple comparisons test: ***P < 0.001, **P < 0.01, *P < 0.05, ns ≥ 0.05, compared with the corresponding control group.

The protective efficacy of the candidate vaccine was evaluated on the basis of antibody levels and survival rates 120 days after primary immunization. C57BL/6N mice (*n* = 6 per group) were randomly assigned to 3 groups: 61 VG-AIV, Al-AIV, and 61 VG-PBS. Immunizations were administered at 21-day intervals following a prime-boost strategy. Serum samples were collected 120 days after booster immunization, and antibody titres were measured by indirect ELISA to assess the humoral immune response in the 61 VG-AIV group. At 120 days post-immunization, the mice were challenged with a lethal dose of Lm XYSN (20 × LD_50_), and survival rates were monitored (Figure 3B).

### Vaccine challenge in sheep

In this study, twelve three-month-old sheep were randomly assigned to two groups (*n* = 6/group). The sheep were subcutaneously administered either 1.5 mL of the 61 VG-AIV or 1.5 mL of 61 VG-PBS at 28-day intervals. Body weight and rectal temperature were recorded on days 0, 7, 14, 21, 28, 35, 42, and 49 post-immunization. On day 28 after the booster, one sheep from each group was randomly selected for pathological examination, and one unvaccinated sheep served as the negative control. The collected organs (heart, liver, spleen, lung, kidney, and brain) were fixed, sectioned, and HE stained at Servicebio Technology Co., Ltd. (Wuhan).

To evaluate the humoral immune response, blood samples were collected on days 7, 21, 35, and 49 after immunization. The serum was separated, and anti-XYSN IgG antibody levels induced by 61VG-AIV were quantified by indirect ELISA. Two groups of sheep (*n* = 6/group) were primed and boosted with either 61 VG-AIV or 61VG-PBS. On day 21 post-booster infection, the sheep were challenged with a lethal dose of the wild-type XYSN strain (1.0 × 10^10^ CFU) via intramuscular injection, and survival was monitored for 21 days. Survival curves were plotted to assess the protective efficacy of 61VG-AIV in sheep.

### Statistical analysis

Statistical analysis and graphing were conducted using Prism 8 software (GraphPad Software). One-way ANOVA was used to compare the means of two or more groups with those of the control group, whereas two-way ANOVA was used to compare the means of two groups by Tukey’s or Sidak’s multiple comparison tests. T tests were used to compare differences between two data groups. The IgG antibody titres (in mice and sheep) and mouse serum IgG antibody subclasses were analysed by two-way ANOVA with Tukey’s multiple comparison test. The antibody titres at 120 days, the histological analysis scores of pathological changes, and the mRNA transcription levels of cytokines in vaccinated mice were analysed by one-way ANOVA with Tukey’s multiple comparison test. Changes in mouse weight and sheep body temperature were compared using unpaired t tests. Additionally, the post-challenge organ bacterial loads of the mice were analysed via unpaired t tests. Survival curves were compared via the log-rank test for survival analysis. Significance levels are indicated as follows: * *P* < 0.05, ** *P* < 0.01, *** *P* < 0.001, and **** *P* < 0.0001. A *P* value greater than 0.05 (*P* > 0.05) was considered not statistically significant (ns).

## Results

### High safety of 61VG-AIV to mice and sheep

To control infectious diseases caused by immunization, vaccine safety, such as monitoring adverse reactions in vaccine recipients, is the primary consideration [24]. C57BL/6N mice were vaccinated with 61 VG-AIV, Al-AIV, or 61 VG-PBS to assess vaccine safety. The mice in all the groups presented a normal coat color, mental state, and food intake. No significant differences in body weight changes were observed between the vaccinated groups and the unvaccinated control group at 30 days after primer-boost vaccination (Figure 1B). In the 61VG-AIV group, moderate redness and swelling at the injection site were observed, and the lesions regressed within 1 to 2 weeks post-vaccination. On the basis of the scoring criteria outlined in Table 2, we assessed tissue samples for signs of necrosis, immune cell infiltration, and structural damage via histopathological analysis of organ sections. The results revealed no differences compared with those of the control group (Figure 2C). No detectable pathological changes, necrosis, hemorrhage, or immune cell infiltration were detected in the liver, brain, kidney, heart, or lung sections of the mice in any of the groups. Similarly, the rectal temperature and weight of the sheep in the 61 VG-AIV and 61 VG-PBS groups were not significantly different (Figures 2B and C). Histopathological analysis revealed that necrosis, haemorrhage, and infiltration of lymphocytes or macrophages were not observed in heart, liver, spleen, lung, kidney, or brain sections of the sheep in the two groups (Figure 2D). These data demonstrated that 61VG-AIV has a favourable safety profile in both mice and sheep, suggesting its suitability for subsequent protective immune response assays.

### 61 VG-AIV induced a robust humoral immune response

Following prime-boost immunization, both the 61 VG-AIV and Al-AIV groups of mice presented significantly elevated Lm XYSN-specific IgG antibody titres compared with the 61VG-PBS group (****, *P* < 0.0001) (Figure 3A). These findings confirm the ability of 61VG-AIV to elicit a potent humoral immune response in mice. Long-term immune protection, a critical parameter for vaccine efficacy, was evaluated on day 120 post-inoculation.

Compared with the Al-AIV group, the 61 VG-AIV group presented significantly greater antibody titres against Lm (titres: 1:68 000 vs. 1:25 600) (*, *P* < 0.05) (Figure 3B). Dynamic changes in sheep antibody titres revealed a gradual increase in Lm-specific IgG levels following vaccination. By day 49 post initial immunization, the titre was significantly greater in the vaccinated group than in the 61 VG-PBS group (****, *P* < 0.0001) (Figure 3C). In the 61 VG-AIV group, the average antibody titre in sheep reached approximately 250,000, indicating a robust humoral immune response. 61 VG-AIV induced robust humoral immune responses in a mouse model but also elicited strong humoral immune responses in sheep.

### 61 VG-AIV enhanced the proinflammatory immune response in mice

**Figure 4:**
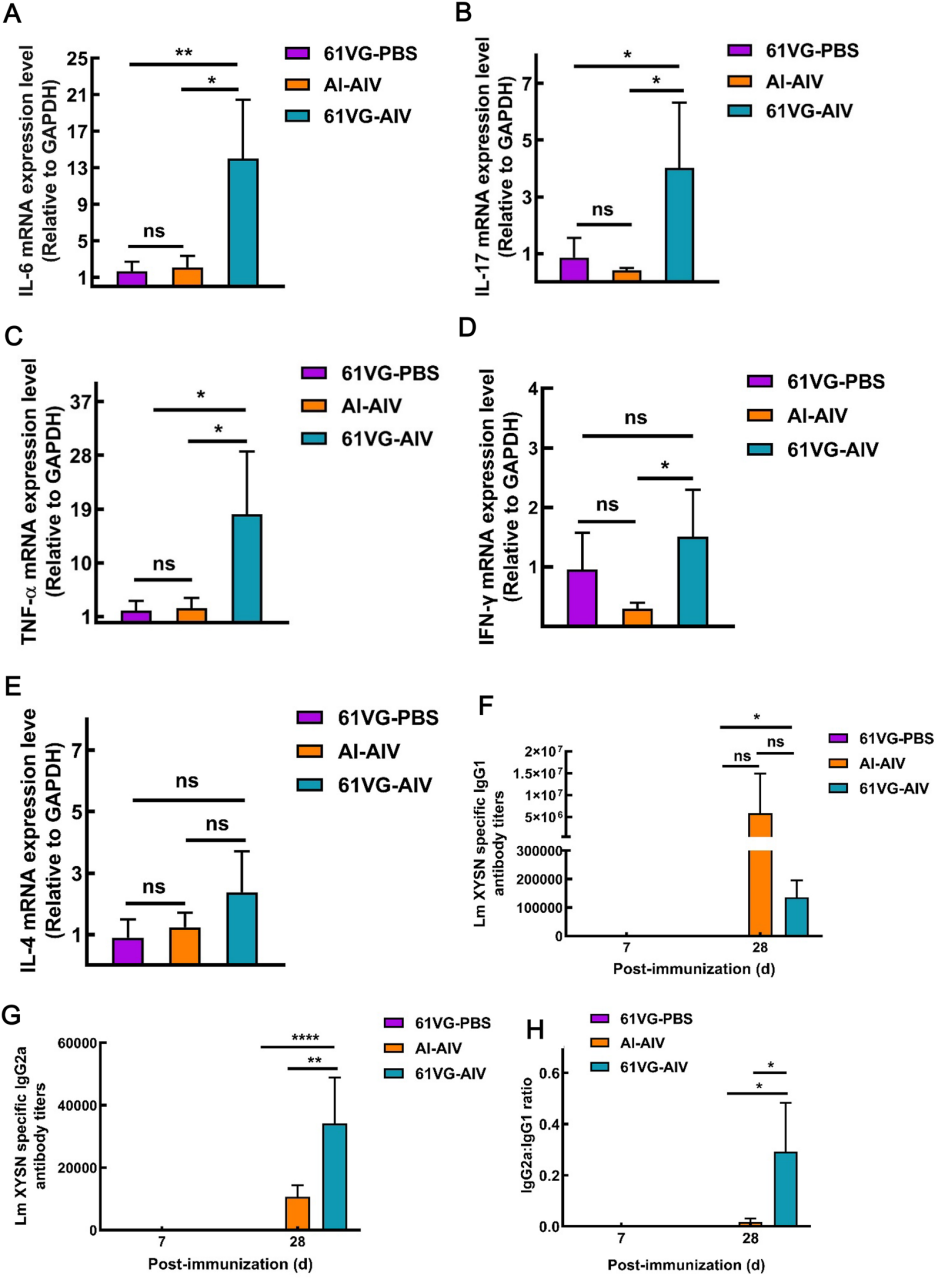
**Immune types of immunized mice**. **A** qRT-PCR transcript levels of IL-6; **B** transcript levels of IL-17; **C** transcript levels of TNF-α; **D** transcript levels of IFN-γ; **E** transcript levels of IL-4; **F** IgG1 antibody titres; **G** IgG2a antibody titres; **H** ratio of IgG2a to IgG1. The error bars represent the SD; *n* = 3 independent experiments. Statistical analyses were carried out via one-way ANOVA followed by Tukey’s multiple comparisons test: ***P < 0.001, **P < 0.01, *P < 0.05, ns ≥ 0.05, compared with the corresponding control group.

Cytokine expression profiles in the host reflect various types of immune responses, which are crucial for assessing the effectiveness of vaccines. The expression levels of proinflammatory and anti-inflammatory cytokines induced by the vaccine candidates were measured by qRT-PCR. The results revealed that the expression of proinflammatory cytokines, including IL-6, IL-17, TNF-α and IFN-γ, was significantly greater in the spleens of the 61 VG-AIV-vaccinated group than in those of the Al-AIV-vaccinated group (Figures 4A, B, C, and D), whereas the transcript levels of the anti-inflammatory cytokine IL-4 were not significantly different (Figure 4E). These results indicate that the 61VG-AIV vaccine induced a stronger proinflammatory response, as evidenced by elevated levels of these cytokines, than both the 61VG-PBS and the Al-AIV. Our data demonstrate that 61VG-AIV induces a robust cellular immune response.

The levels of IgG1 and IgG2a antibodies in mice serve as indicators of Th2- and Th1-type immune responses, respectively [25]. After booster immunization, the IgG1 antibody titre in the mice vaccinated with 61 VG-AIV was not significantly different from that in the mice vaccinated with Al-AIV (Figure 4F). However, the IgG2a antibody titres and the IgG1/IgG2a ratio in the 61 VG-AIV group were significantly greater than those in the Al-AIV group (Figures 4G and H). These results demonstrated that the ISA 61 VG adjuvant enhances a stronger Th1-biased immune response to inactivated Lm XYSN than does the aluminum adjuvant.

### 61 VG-AIV protected against lethal challenge with the serotype 4h Lm

**Figure 5:**
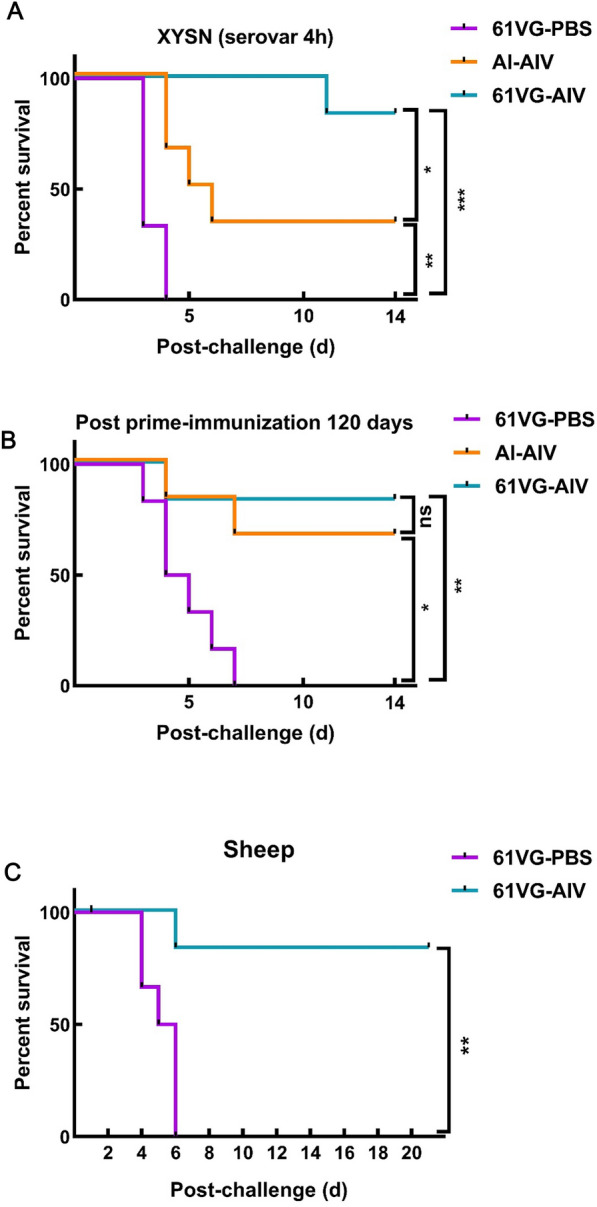
**Survival curves of vaccinated animals after lethal challenge. Following lethal challenge, the percent survival of different Lm serotypes was determined**. **A** Survival curves of XYSN strain-challenged mice from different groups at 4 h. **B** Survival curves of mice from different groups at 120 days post-booster immunity challenge with Lm XYSN (*n* = 6 mice per group; all the mice were euthanized on day 14 post-infection). **C** Survival curves of sheep from the two groups at 21 days after the Lm challenge booster (*n* = 6 sheep per group; all sheep were euthanized on day 21 post-infection). The error bars represent the SD; *n* = 3 independent experiments. Statistical analyses were carried out via the log-rank test for survival analysis: ****P < 0.0001, ***P < 0.001, **P < 0.01, *P < 0.05, ns ≥ 0.05.

The protective efficacy of 61 VG-AIV in a mouse model was evaluated through primer-boost vaccination and challenge with a lethal dose of wild-type Lm XYSN via oral inoculation. The results showed that 61 VG-AIV provided significant protection, with an 83.4% survival rate observed in mice challenged with a lethal dose (50 × LD_50_) of Lm XYSN. This marked a notable improvement compared with the Al-AIV group, which achieved a 33.4% protection rate (Figure 5A). To further evaluate immune protection, a critical parameter for vaccine efficacy, the immune protection rate was assessed on day 120 post-inoculation. After oral challenge with the wild-type Lm XYSN strain, the 61 VG-AIV group presented an immune protection rate of 83.4% against a lethal dose (20 × LD_50_). In comparison, the Al-AIV group achieved a 66.7% protection rate, whereas the 61 VG-PBS group showed no protection (Figure 5B). Our findings suggest that the antibody levels induced by the 61 VG-AIV vaccine persist up to 120 days post-immunization. These results indicate that the 61VG-AIV vaccine elicits a strong immune response and provides valuable insights into the duration of immune protection offered by the inactivated vaccine.

**Figure 6:**
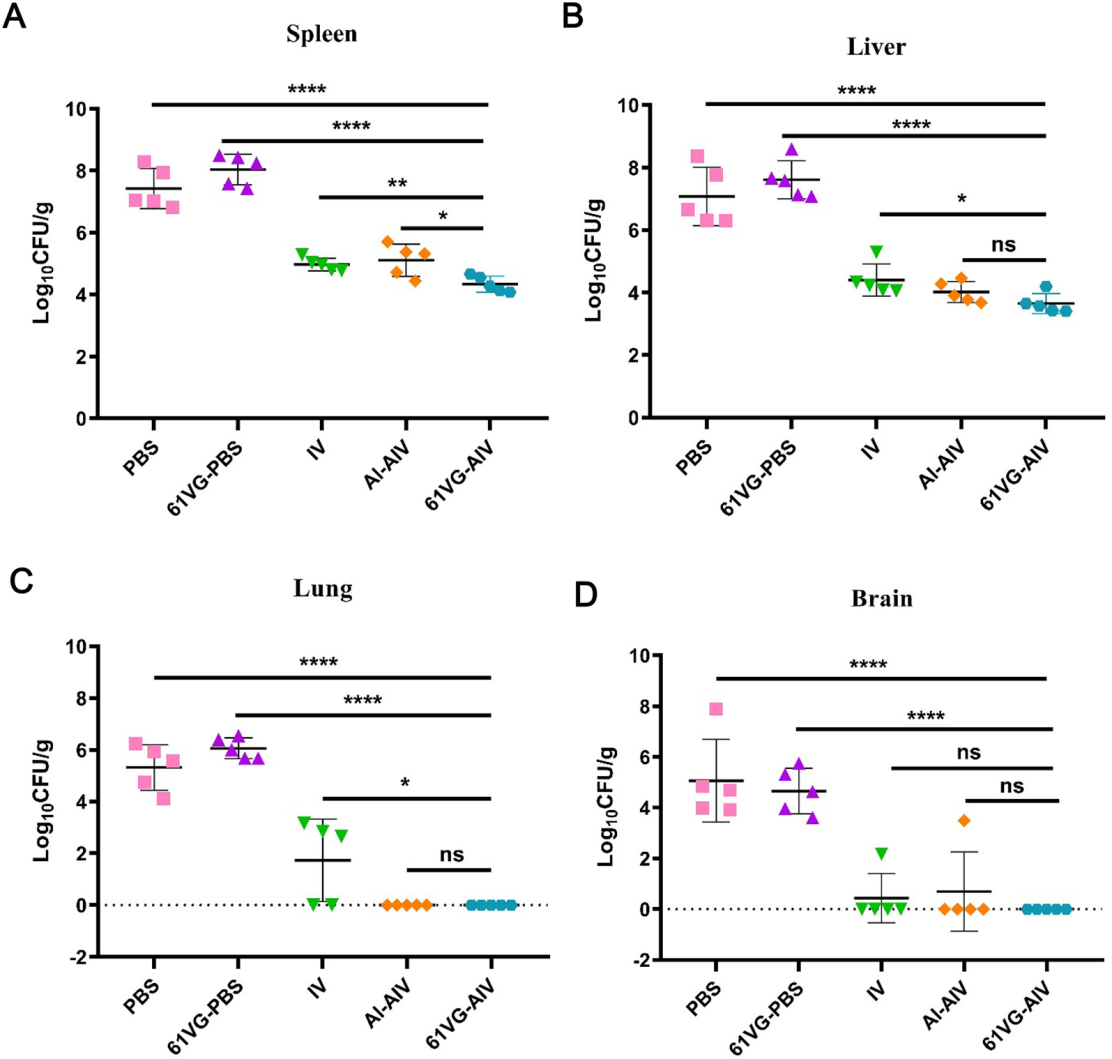
**Comparison of the organs bacterial load among C57BL/6N mice in different inoculation groups after XYSN challenge**. **A–D** At 4 days post-infection with the XYSN strain, the log CFU/g of spleen, liver, lung, and brain tissues were collected from five groups of C57BL/6N mice, representing the mean value for each group of 5 mice, with each point corresponding to the organ of one infected mouse. Statistical analysis was performed by one-way ANOVA followed by Tukey’s multiple comparisons test: ****P < 0.0001, ***P < 0.001, **P < 0.01, *P < 0.05, ns ≥ 0.05.

**Figure 7:**
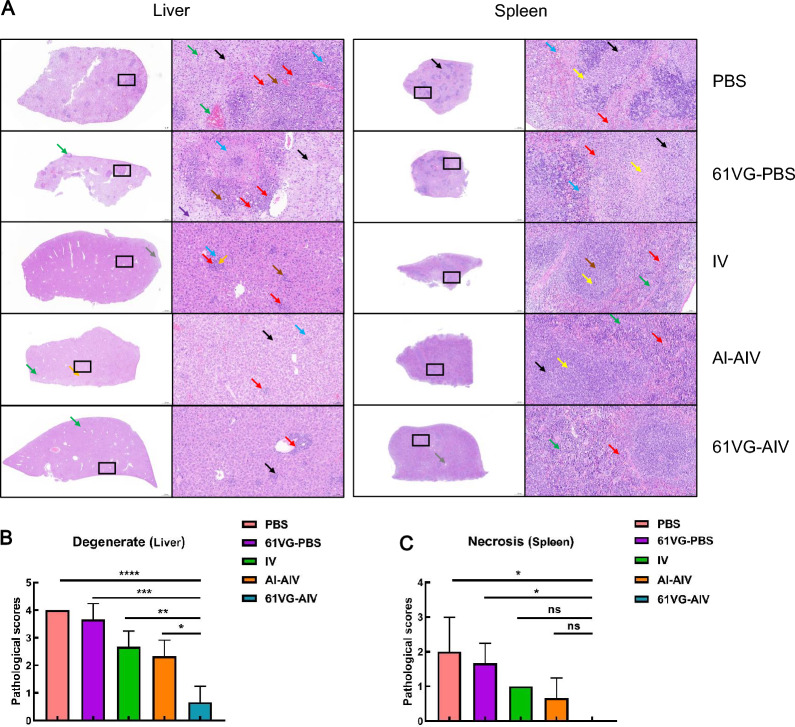
**Pathological changes in organs of C57BL/6N mice in different inoculation groups after XYSN challenge**. **A** The red arrow in the HE-stained section micrograph of the mouse liver shows lymphocyte infiltration; blue indicates cell necrosis; brown indicates connective tissue hyperplasia; orange represents extramedullary hematopoietic cells; gray represents hydropic degeneration; green represents venous congestion; purple represents balloon-like degeneration; and the black arrow represents hepatic steatosis. The red arrow in the HE-stained micrograph of mouse spleen slices shows granulocyte infiltration; yellow represents cell necrosis; green represents extramedullary hematopoietic cells; brown represents the expansion of the white pulp germinal center; black represents connective tissue hyperplasia. The gray arrows in the HE-stained micrograph of the mouse lung slices represent necrotic cell fragments; yellow represents bleeding; green represents venous congestion; black represents infiltration of granulocytes; blue represents eosinophilic mucus secretion; light green represents insoluble fibrin deposition; gray represents epithelial cell necrosis; brown represents connective tissue hyperplasia; purple represents alveolar dilation; and orange represents edema. The scale for the low-magnification field of view in the image is 500 μm (20 × magnification), and the high-magnification field of view is 50 μm (200 × magnification), as shown in the black box. **B** Pathological scoring results for liver cell degeneration in mice. **C** Pathological scoring results for spleen cells and tissue necrosis in mice. The error bars represent the SD; *n* = 3 independent experiments. Statistical analyses were carried out via one-way ANOVA followed by Tukey’s multiple comparisons test: ****P < 0.0001, ***P < 0.001, **P < 0.01, *P < 0.05, ns ≥ 0.05.

On day 4 post-challenge with wild-type Lm XYSN, the bacterial loads in the spleens of the 61 VG-AIV group were significantly lower than those in the spleens of the Al-AIV, IV, PBS and 61 VG-PBS groups (Figure 6A). In addition, the bacterial loads in the livers, lungs and spleens of 61 VG-AIV group mice were also significantly lower than those in the other groups (IV, PBS or 61 VG-PBS groups) (Figures 6A, B, C, and D). Histopathological analysis revealed that liver tissue damage in mice vaccinated with 61 VG-AIV was less severe than that in the other groups, which presented tissue necrosis, degeneration, and connective tissue proliferation (Figure 7A, B and C) (Additional file 1). Similarly, cell necrosis and connective tissue proliferation in the spleens of the 61 VG-AIV immunization group were significantly lower than those in the PBS or 61 VG-PBS groups (Additional file 2). These data indicate that 61 VG-AIV immunization can reduce bacterial colonization ability in organs such as the liver and spleen and alleviate organ damage caused by Lm XYSN challenge.

Ruminant animals, known for their susceptibility to Lm infection, underscore the importance of *Listeria*-based vaccines in effectively preventing and controlling animal listeriosis. This study aimed to evaluate the protective immune responses elicited by 61 VG-AIV in a sheep model. The survival of the sheep was monitored after challenge with 1.0 × 10^10^ CFU of Lm XYSN, and survival curves were plotted (Figure 5C). In the 61 VG-PBS sheep, successive deaths occurred between day 4 and day 6, resulting in a protection rate of 0%. In contrast, the 61 VG-AIV sheep exhibited a survival rate of 83.4%, highlighting the ability of 61 VG-AIV to provide robust immune protection against lethal challenges with Lm XYSN. These trial results emphasize the superior immune protection of 61 VG-AIV when faced with Lm XYSN. These findings underscore the enhanced immune response and protective efficacy of the 61 VG-AIV vaccine against lethal challenges with Lm XYSN in mice and sheep.

### 61 VG-AIV conferred cross-protection against challenge with three serotypes of Lm in mice

**Figure 8:**
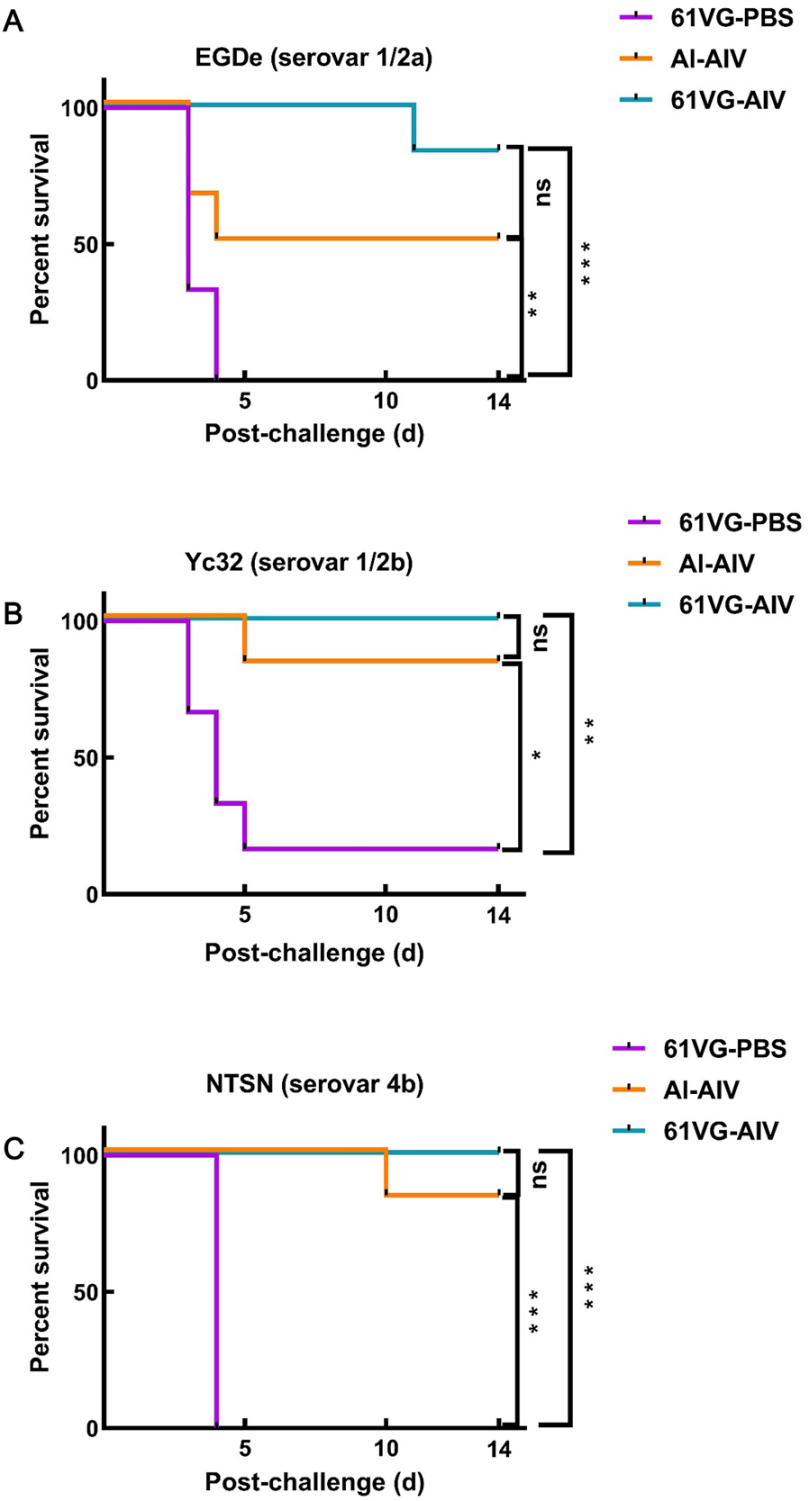
**Cross-immunoprotection by 61 VG-AIV against various serotypes of Lm in mice. The percent survival of challenges against different serotypes of Lm**. **A** Survival curve of serotype 1/2a EGD-e strain-challenged mice. **B** Survival curve of serotype 1/2b Yc32 strain-challenged mice. **C** Survival curve of serotype 4b NTSN strain-challenged mice (*n* = 6/group; all the mice were euthanized on day 14 post-infection). The error bars represent the SD; *n* = 3 independent experiments. Statistical analyses were carried out via the log-rank test for survival analysis: ****P < 0.0001, ***P < 0.001, **P < 0.01, 0.01 < *P < 0.05, ns ≥ 0.05.

The efficacy of *Listeria* vaccines based on a single serotype strain has been well documented from multiple groups working over the past decades. However, research on cross-serotype immune protection remains challenging because of the heterogeneity of somatic antigens and H antigens across the 14 Lm serotypes. In this study, we assessed the potential cross-protective ability of 61 VG-AIV in a mouse model by challenging with serotypes 1/2a, 1/2b, and 4b Lm. Notably, the 61 VG-AIV group exhibited greater levels of protection against the 1/2a strain (83.4% vs. 50%), the 1/2b strain (100% vs. 83.4%), and the 4b strain (100% vs. 83.4%). In contrast, the 61 VG-PBS group showed 0% protection, except against the Yc32 strain, where the protection rate was 16.7% (Figures 8A, B, and C). These findings demonstrate that 61 VG-AIV induces cross-serotype protection against 1/2a, 1/2b, and 4b Lm infections, surpassing the immune protection conferred by Al-AIV.

## Discussion

Although traditional inactivated vaccines are widely used for preventing infectious diseases because of their good safety profile, they often exhibit limited immunogenicity. Adjuvants play a crucial role in overcoming this limitation by enhancing both cellular and humoral immune responses. Previous studies have established the safety and enhanced immunogenicity of 61 VG-AIV in mammalian models, which have been specifically adapted for ruminant vaccine applications [12, 26–28]. In this study, the Montanide™ ISA 61 VG water-in-oil adjuvant was used to potentiate the inactivated Lm XYSN vaccine. The safety profiles of ISA 61 VG adjuvanted vaccine were evaluated in both murine and ovine subjects, which demonstrated promising safety credentials. Additionally, 61 VG-AIV confers protection against Listerial infections in both mouse and sheep models. These findings underscore the potential of ISA 61 VG as an effective adjuvant for enhancing vaccine-induced immune responses and expanding its protective efficacy against various *Listeria* serotypes.

Inactivated vaccines generally have good safety profiles, but they face challenges due to their limited immunogenicity; therefore, the development of novel adjuvants aims at enhancing Th1-type biased responses [29]. Th1-type immune responses are particularly involved in cellular immunity and the inflammatory response. Th1 cells enhance the adaptive immune response and fight against intracellular pathogens by secreting cytokines such as IFN-γ, IL-6, IL-17, and TNF-α. Th2-type immune responses are associated with humoral immunity and play critical roles in resisting extracellular pathogen infection. Th2 cells primarily secrete anti-inflammatory cytokines such as IL-4, promoting the generation of antibody subclasses such as IgG1 in mice, which are involved in humoral responses [30]. The traditional alum-adjuvanted inactivated *Listeria* vaccine induces a Th2-biased immune response, resulting in limited efficacy against Lm infection [31]. Our study revealed that, compared with Al-AIV, 61 VG-AIV induced significantly higher IgG2a antibody levels and an increased IgG2a/IgG1 ratio in mice. These findings suggested that compared with the aluminium adjuvant, 61 VG-AIV elicited a stronger Th1-biased immune response. Our results demonstrated that 61 VG-AIV elicited significantly increased transcript levels of IL-6 and IL-17. Additionally, we assessed the levels of TNF-α, a key indicator of Th1-mediated cellular immune responses [32, 33]. Elevated TNF-α levels in mice vaccinated with 61 VG-AIV indicate an increase in Th1-mediated cellular immune responses. IL-4 is an anti-inflammatory cytokine produced during infections, with IL-4 considered a hallmark cytokine of Th2-type immune responses [34]. In our study, no significant changes in the IL-4 transcript levels were observed. Humoral immune responses play a critical role in neutralizing and eradicating Lm within the host, as shown by previous studies [23, 24]. Our findings indicated that the incorporation of 61 VG-AIV significantly enhanced the humoral immune response to the inactivated vaccine in both mice and sheep, thereby increasing overall efficacy. Th1 cells mediate the production of antibody subclasses such as IgG2a in mice, which is related to cell-mediated immunity.

Lm comprises 14 serotypes, among which 1/2a, 1/2b, and 4b strains are responsible for more than 95% of listeriosis outbreaks [21]. Conventional Lm vaccines generally protect against strains of the same serotype [35]. Thus far, the protective efficacy of combined attenuated vaccine candidates have rarely been reported in mouse and sheep models. The use of multivalent *Listeria* vaccines complicates the preparation process and increases production costs. Lm XYSN, which belongs to serotype 4 h, shares a somatic antigen with serotype 4b and a flagellar antigen with serotype 1/2a strains, which suggests potential cross-serotype immune protection. Our prior live attenuated serotype 1/2b Lm vaccine has shown cross-protection against serotypes 1/2b and 4b Lm [36], yet no inactivated vaccine has been reported to have such broad efficacy until now. This study demonstrated that 61 VG-AIV can confer high protection efficacy against serotypes 1/2a, 1/2b, and 4b and 4 h of Lm challenge, indicating that 61 VG-AIV can confer expanded serotype cross-protection due to the unique O antigen and H antigen combination of Lm XYSN. Our study provides new insights into strategies for developing novel vaccines against infections caused by multiple predominant Lm serotypes.

The primary goal in vaccine development is to achieve enduring and effective protection. Therefore, assessing a vaccine's efficacy depends on its capacity to induce lasting immune protection [37]. Prior studies have revealed a decline in mouse resistance to systemic listeriosis infection 60–120 days after a single immunization with a vector vaccine [38]. Lauvau et al. demonstrated that immunization with heat-killed *Listeria monocytogenes* (HKLM) primes T lymphocytes to form memory CD8^+^ T lymphocyte populations crucial for long-term protective immunity, although HKLM fails to effectively prevent subsequent Lm infection [39]. In contrast, compared with Al-AIV, 61 VG-AIV exhibited superior immune protection even at 120 days post-immunization. These findings illustrateed that, compared with Al-AIV, 61 VG-AIV provides stronger cross-protective immunity against challenges with multiple Lm serotypes. Moreover, it protected more than 80% of the immunized mice for up to 4 months, indicating remarkable long-term cross-protection. Nevertheless, further research is warranted to explore the durability and extent of long-term immunity conferred by 61 VG-AIV in natural hosts, contributing to a more comprehensive understanding of its potential as a vaccine candidate.

Sheep and cattle are recognized as natural reservoirs susceptible to Lm [40]. However, vaccine candidates have rarely been evaluated in ovine mode. The live attenuated vaccine developed by Linde et al. showed an immune protection rate of 71.9% in vaccinated sheep, compared with 28.1% in the control group [18]. Additionally, another study showed that vaccination with live attenuated vaccines could reduce the incidence of listeriosis by 2.5% in sheep [20]. Notably, live attenuated Lm vaccines pose inherent risks of adverse effects and systemic infections [20, 41]. To date, no reports exist on *Listeria*-inactivated vaccines. Although ISA 61 VG has been studied in ruminants [16], its application as an adjuvant in listeriosis vaccines for ruminants is unsubstantiated. In a study by Linde et al., a bivalent attenuated vaccine (1/2a and 4b) significantly improved immune protection efficiency in sheep by 43.8% compared with the control group [18]. In our prior study, a live attenuated Lm vaccine resulted in a 78% improvement in the immune protection rate in sheep compared with the PBS group [19]. In the present study, 61 VG-AIV conferred an 83.4% protection rate in sheep following a lethal Lm XYSN challenge compared with the 61 VG-PBS group. These findings suggested that compared with live attenuated vaccines, 61 VG-AIV offers superior immune protection in sheep while maintaining greater safety.

In conclusion, this study demonstrates that the 61 VG-AIV vaccine is safe in both mouse and sheep models. The vaccine candidate induced a strong humoral immune response in both mice and sheep and a Th1-biased immune profile in mice, showing cross-protective immunity against infection by four predominant Lm serotypes. Our data provide new insights into the development of vaccines against infections caused by multiple serotypes of pathogens. However, large-scale animal trials and field application validations remain necessary in the next steps.

## Supplementary Information

**Additional file 1. Liver lesion scores of the mice after challenge**.

**Additional file 2. Spleen lesion scores of the mice after challenge**.

## Data Availability

All the data generated or analysed during this study are included in this published article and its supplementary information files.
