# Supplementary material for: The inactivated and ISA 61 VG adjuvanted vaccine enhances protection against cross-serotype Listeria monocytogenes
Source: Vet Res. 2025 Mar 20;56:60. doi: 10.1186/s13567-025-01483-2 (PMC11924870; doi:10.1186/s13567-025-01483-2)
Supplement: Supplementary file 2 — Additional file 2. Spleen lesion scores of the mice after challenge. [file 13567_2025_1483_MOESM2_ESM.docx]

**Additional file 2** **Spleen lesion score of mice after challenge**

| **Groups** | **Connective tissue hyperplasia (0-4)** | **Necrosis**  **(0-4)** | **Inflammatory cell infiltration (0-4)** | **Extramedullary hematopoietic cells (0-4)** | **Expansion of hair growth center (0-4)** |
| --- | --- | --- | --- | --- | --- |
| **PBS** | 2.33 ± 0.58 | 2 ± 1.00 | 1.33 ± 0.58 | 0.00 ± 0.00 | 0.00 ± 0.00 |
| **PBS+61VG** | 4.00 ± 0.00 | 2.33 ± 0.58 | 1.00 ± 0.00 | 0.00 ± 0.00 | 0.00 ± 0.00 |
| **IV** | 0.00 ± 0.00 | 1 ± 0.00 | 1.00 ± 0.00 | 1.67 ± 1.15 | 1.00 ± 1.00 |
| **Al-AIV** | 0.00 ± 0.00 | 0.67 ± 0.58 | 1.33 ± 0.58 | 2.67 ± 0.58 | 0.67 ± 0.58 |
| **61VG-AIV** | 0.00 ± 0.00 | 0.00 ± 0.00 | 2.00 ± 0.00 | 4.00 ± 0.00 | 0.33 ± 0.58 |
